# Analysis of ependymal ciliary beat pattern and beat frequency using high speed imaging: comparison with the photomultiplier and photodiode methods

**DOI:** 10.1186/2046-2530-1-8

**Published:** 2012-06-07

**Authors:** Chris O'Callaghan, Kulvinder Sikand, Mark A Chilvers

**Affiliations:** 1 Department of Infection, Immunity and Inflammation, Division of Child Health, CSB, University of Leicester, Leicester, LE2 7LX, UK; 2 Respiratory Medicine, Portex Unit, Institute of Child Health, University College London (UCL) and Great Ormond Street Hospital, 30 Guilford Street, London, WC1N 1EH, London, UK; 3 Division of Pediatric Respiratory Medicine, BC Childrens Hospital, 4480 Oak Street, Vancouver, V6H 3V4, BC, Canada

**Keywords:** Cilia, Ependymal cilia, CSF, Ciliary beat frequency

## Abstract

**Background:**

The aim of this study was to compare beat frequency measurements of ependymal cilia made by digital high speed imaging to those obtained using the photomultiplier and modified photodiode techniques. Using high speed video analysis the relationship of the power and recover strokes was also determined.

**Methods:**

Ciliated strips of ependyma attached to slices from the brain of Wistar rats were incubated at 30°C and observed using a ×50 water immersion lens. Ciliary beat frequency was measured using each of the three techniques: the high speed video, photodiode and photomultiplier. Readings were repeated after 30 minutes incubation at 37°C. Ependymal cilia were observed in slow motion and the precise movement of cilia during the recovery stroke relative to the path travelled during the power stroke was measured.

**Results:**

The mean (95% confidence intervals) beat frequencies determined by the high speed video, photomultiplier and photodiode at 30°C were 27.7 (26.6 to 28.8), 25.5 (24.4 to 26.6) and 20.8 (20.4 to 21.3) Hz, respectively. The mean (95% confidence intervals) beat frequencies determined by the high speed video, photomultiplier and photodiode at 37°C were 36.4 (34 to 39.5), 38.4 (36.8 to 39.9) and 18.8 (16.9 to 20.5) Hz. The inter and intra observer reliability for measurement of ciliary beat frequency was 3.8% and 1%, respectively. Ependymal cilia were observed to move in a planar fashion during the power and recovery strokes with a maximum deviation to the right of the midline of 12.1(11.8 to 13.0)° during the power stroke and 12.6(11.6 to 13.6)° to the left of the midline during the recovery stroke.

**Conclusion:**

The photodiode technique greatly underestimates ciliary beat frequency and should not be used to measure ependymal ciliary beat frequency at the temperatures studied. Ciliary beat frequency from the high speed video and photomultiplier techniques cannot be used interchangeably. Ependymal cilia had minimal deviation to the right side during their power stroke and to the left during the recovery stroke.

## Background

A single cell layer of ependymal cells separates the cerebrospinal fluid (CSF) of the ventricular system from underlying neuronal tissue. Their biology and pathology have been reviewed in detail by Del Bigio [1]. Ependymal cells have approximately forty rapidly beating cilia that move CSF immediately adjacent to the surface of the brain [2,3].

Their precise role is unclear but it has been suggested that they may have a role in host defence keeping pathogens and debris away from the surface of the brain and by rapidly moving CSF immediately adjacent to the ependymal surface to facilitate diffusion of toxins from the brain into the CSF. Recent data support their role in creating concentration gradients to facilitate neuroblast migration [4]. Abnormalities of ependymal ciliary function due to genetic defects seen in primary ciliary dyskinesia or secondary to infection or toxic insults may result in hydrocephalus [5-12].

As ependymal cilia beat at approximately twice the frequency of respiratory cilia [13] we were keen to evaluate different methods commonly used to measure ciliary beat frequency.

The methods that have been used most frequently to measure ciliary beat frequency are the photodiode, the photomultiplier, cinematography and more recently, digital high speed video recording. The photodiode method involves displaying the images of moving cilia, captured by a video camera, on a monitor. A photodiode cell is positioned on the monitor over the image of the moving cilia. The variation in light intensity resulting from ciliary movement is detected by the photodiode and a signal generated which may be displayed on an oscilloscope [14] or processed by computer to give a power spectrum analysis [15]. The photomultiplier technique, initially described by Dalhamn [16], has been widely used to measure ciliary beat frequency [17,18]. Variations in the perpendicular light beam of the microscope, caused by beating cilia, are detected by a photosensitive cell. Voltage signals generated may then be displayed on an oscilloscope or a power spectrum analysis performed allowing frequency to be obtained. However, both the photodiode and photomultiplier techniques are subject to vibration artefact and neither allow evaluation of the ciliary beat pattern. This is important as cilia may maintain their beat frequency despite beating in a dyskinetic pattern [19,20].

Cinephotographic techniques were regarded as the gold standard for the measurement of ciliary beat frequency [21]. These methods allowed cilia to be recorded at high frame rates, up to 500/second, and then analyzed by replaying in slow motion which allows both the ciliary beat frequency to be determined and the precise beat pattern of individual cilia to be evaluated. Although recognized to be the most accurate method [16,22-24] it was technically difficult and real time analysis was not possible due to film processing. Over recent years, high speed digital video cameras have been developed, allowing recordings to be made at frame rates up to 45,000 per second. High speed digital video cameras provide a simple and accurate method for determining ciliary beat frequency [25] and beat pattern of individual cilia may be seen and analyzed in slow motion following recording [19,20,26]. There have been no direct comparisons of the use of the photodiode, photomultiplier and high speed video techniques to measure the beat frequency of ependymal cilia.

It was thought for many years that respiratory cilia had a forward power stroke and recovery stroke during which the cilium swept backwards and to the side, an action believed to stimulate adjacent cilia, propagating the ciliary metachronal wave [27]. However, using slow motion replay from high speed video recordings we found that human respiratory cilia beat forwards and backwards within the same plane without a classical sideways recovery sweep [28]. We hypothesized that despite propelling fluid as opposed to mucus and being longer and beating faster than respiratory cilia [13], ependymal cilia would also beat forwards and backwards in the same plane without a sideways recovery sweep. In a recent study Lechtreck and colleagues studied the movement of ependymal cilia using high speed recordings and commented that they had a relatively planar back and forth motion. This was not formally measured [29].

The primary aim of this study was to compare the measurement of ependymal ciliary beat frequency made by the photodiode, photomultiplier and digital high speed video methods. A secondary aim was to use digital high speed video imaging to determine the precise relationship between the forward power and backward recovery stroke of fluid propelling ependymal cilia.

## Methods

### Preparation of brain ependymal tissue

We have previously described methods used for sample preparation and beat frequency analysis [30,31]. Wistar rats (9 to 15 days of age) were sacrificed by cervical dislocation. The brain was immediately removed and stored in medium 199 (pH7.4; plus penicillin 50 u/ml and streptomycin 50 μg/ml) at 4°C until use. The fourth ventricle was chosen as the most reliable site for the presence of cilia. The brainstem and cerebellum were separated from the cerebral hemispheres and blood vessels and meninges removed.

To decrease trauma to the fragile ependyma caused by manual slicing, a vibratome was used to cut brain slices. The brainstem was mounted, aqueduct side down, on the cutting stage of a Vibroslice (Model 752: Campden Instruments Ltd). The bath surrounding the brain stem was filled with medium 199 and cooled to 4°C using a Peltier driven cooling device. Transverse slices of brain, 150 μm thick, were cut and stored in medium 199 at 4°C (pH7.4: plus penicillin 50 u/ml and streptomycin 50 μg/ml) until use. Ependyma from the fourth ventricle of 10 to 15 day old rats was used. For each piece of tissue associated with the fourth ventricle, approximately two to three ciliated brain slices with intact ependymal edges were obtained. Ciliated ependyma which had obviously been disrupted or damaged was not used.

For the experiment, brain slices were mounted in a well containing 4 ml of medium 199 with Earle's sales (pH 7.4: plus penicillin 50 u/ml and streptomycin 50 μg/ml). The well was placed in a purpose built environmental chamber which was thermostatically controlled to keep the fluid surrounding the ependymal sample at either 30°C or 37°C. The chamber was humidified to 75% to 80% to prevent evaporation from the well during the study period.

Three techniques were used to measure ciliary beat frequency: the high speed video; photomultiplier; and photodiode. These are described in greater detail below. The study involved incubation of the ependymal samples at 30°C for 30 minutes followed by initial measurements, using each of the three techniques. Samples were then incubated at 37°C for a further 30 minutes and readings repeated, again using the three methods. The order in which the various methods of measurement were made was varied from experiment to experiment to help exclude any bias due to their order of use.

Ciliary movement was observed using a ×50 water immersion lens. The maximum time the tissue was in focus was between 30 and 45 seconds for each reading.

In total, 28 separate experiments on different ependymal strips were conducted at both 30°C and 37°C. The photodiode was not available for use in all of the experiments (n = 16 at 30°C and n = 23 at 37°C) due to technical problems.

The following section describes the methods used to measure ciliary beat frequency.

### High speed video

Beating ependymal ciliated strips were recorded by a high speed video camera (Kodak EktaPro Motion Analyser, Model 1012) at a rate of 400 frames per second using a shutter speed of 1 in 2,000 as previously described [30,31]. The camera allowed video sequences to be downloaded at reduced frame rates, allowing ciliary beat frequency to be determined directly by timing a given number of individual ciliary beat cycles. At each time point of the study, ciliary beat frequency was measured at four different areas along each ependymal strip. Ciliary beat frequency (CBF) may be determined directly by timing a given number of individual ciliary beat cycles. Groups of beating cilia were identified and the number of frames required to complete 10 beat cycles was recorded. This was converted to CBF by a simple calculation (CBF = 400/(number frames for 10 beats) × 10). Only intact, undisrupted, ciliated strips in excess of 100 μm were studied. Using this method, it is also possible to observe the precise movement of individual cilia during their beat cycle.

### Photodiode measurements

Video images of the beating ependymal cilia were relayed from an S-VHS video camera (Panasonic VW15) to a high resolution monitor. The photodiode, mounted in a pen like system, was held over the beating cilia on the monitor. Voltage signals were generated as cilia moved past the photodiode sensor and were fed via an oscilloscope to a power spectrum analysis program (ANADAT, Montreal, Canada) to determine ciliary beat frequency. The frequency of cilia was measured at four separate places along the ciliated edge allowing a mean value of ciliary beat frequency to be obtained.

### Photomultiplier measurements

The aperture allowing light to reach the photomultiplier was adjusted to 2 μm^2 ^and positioned over an area of beating cilia. Voltage signals generated were then displayed on an oscilloscope and relayed to the power spectrum analysis program (ANADAT, Montreal, Canada) to determine ciliary beat frequency.

The frequency of cilia was measured at four separate places over the ciliated edge allowing a mean value of ciliary beat frequency to be obtained.

### Inter and intra observer variability using the digital high speed video system

Following the results of the comparison between methods, the high speed video system was selected as the method of choice for ependymal ciliary experiments. The reproducibility of the technique was evaluated by establishing inter and intra observer variability in measurements.

Ten ciliated edges from different rats were incubated at 37°C for at least 30 minutes and a high speed video recording made. This was stored on video tape at a reduced frame rate for later analysis.

An observer measured beat frequency by determining the time taken for individual cilia to complete 5, 10 and 15 beat cycles. The edge from which readings were made was divided into quadrants and the beat frequency of an individual cilia from each quadrant was determined. Thus, the effect of counting different beat cycles using different ciliated edges and taking measurements from different places along a given edge could be assessed. Measurements were made again by the same observer one month later using the archived video recording.

To compare the variation in beat frequency measurement between observers, a second observer independently measured beat frequency from the same ten edges. Frequency was measured by determining the time taken for individual cilia to complete ten beat cycles. As with the first observer, the edge was divided into quadrants and a reading taken from each quadrant.

### Beat pattern analysis

The experimental system allowed the beating cilia to be viewed beating directly towards the observer.

Beating ciliated edges were recorded using a digital high speed video camera (Kodak Ektapro Motion Analyser, Model 1012) at a rate of 400 frames per second, using a shutter speed of 1 in 2000. The camera allows video sequences to be recorded and played back at reduced frame rates or frame by frame. The precise movement of individual cilia was observed during the complete beat cycle.

The precise path taken by a cilium during the power and recovery strokes was evaluated by modifying a previously described method [28]. Viewing the cilia beating towards the observer, the precise position of the cilium during the forward power stroke was plotted frame by frame on an acetate sheet overlying the high resolution monitor. As the cilium moved backward during the recovery stroke its position during this movement was again plotted frame by frame.

A total of six rat brains were evaluated and a minimum of four ependymal epithelial edges were examined for each brain.

**Figure 1:**
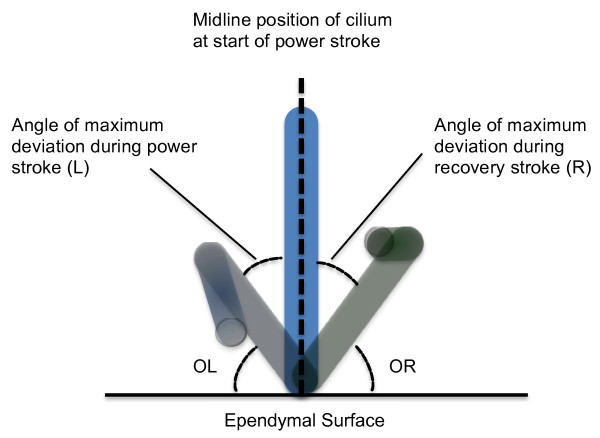
**Angles measured when determining ciliary beat pattern**. A horizontal line was drawn along the ependymal edge and a vertical line drawn through the cilium at the start of the power stroke in the midline position. Frame by frame analysis allowed the precise beat pattern to be viewed. The diagram illustrates the maximum deviation of the cilium during the power stroke (light blue cilium) and the maximum deviation of the cilium during the recovery stroke (light green cilium). The following angles were measured: OL: Outer Left: angle between the horizontal and the maximum displacement of the cilium to the left of midline. L: angle of maximum deviation of cilium left of midline. R: angle of maximum of cilium right of midline. OR: Outer Right: angle between the horizontal and the maximum displacement of the cilium to the right of midline.

Figure 1 illustrates the different measurements recorded. A horizontal line was drawn along the ependymal edge and a vertical line drawn through the cilium at the start of the power stroke in the midline position. The following angles were measured:

- OL: Outer Left: angle between the horizontal and the maximum displacement of the cilium to the left of midline.

- L: angle of maximum deviation of the cilium left of midline

- R: angle of maximum deviation of the cilium right of midline

- OR: Outer Right: angle between the horizontal and the maximum displacement of the cilium to the right of midline.

For each cilium studied the angle for five complete beat cycles was measured by image analysis (Scion image, Scion Corporation, Frederick, Maryland, US).

### Statistics

A two way analysis of variance was performed with factors: 1) Temperature: 37°C or 30°C and 2) Method: HSV, Photo, Diode.

As the difference between the methods varies with temperature, the limits of agreement were calculated for each temperature and each method separately. The high speed video method is considered to be the gold standard. Therefore, the mean and standard deviation of the difference between each method and the high speed video at each temperature was calculated. Paired t tests were performed to compare each method with the high speed video method. The Bland and Altman limits of agreement were calculated from the mean difference ± twice the standard deviation of the differences [32].

#### Intra subject variation in the measurement of ciliary beat frequency

An analysis of variance was performed using the restricted maximum likelihood estimation (REML) procedure (Genstat) to estimate the variance components. There were four factors: edge number (10); beat cycle (5, 10, 15); quadrant (4 places); occasion (1 and 2). All four factors were considered to be random. The various components for each main effect and all combinations of two factors were estimated. The analysis was then repeated after omission of any variance components from the model which had negative estimates. Separate analyses were then performed 5, 10 and 15 cycles.

#### Inter observer variability in ependymal ciliary beat frequency using high speed video

there were two observers. Each observer performed four readings at ten different places along the ciliated strip of epithelium. Analysis of variance was performed considering the two observers to have fixed effects. The variance components were estimated using the REML procedure in Genstat [33].

The mean, standard deviation, and 95% confidence intervals were calculated for the angles (OL, OR, L, R). Paired t tests were performed comparing OL with OR and L with R.

## Results

**Figure 2:**
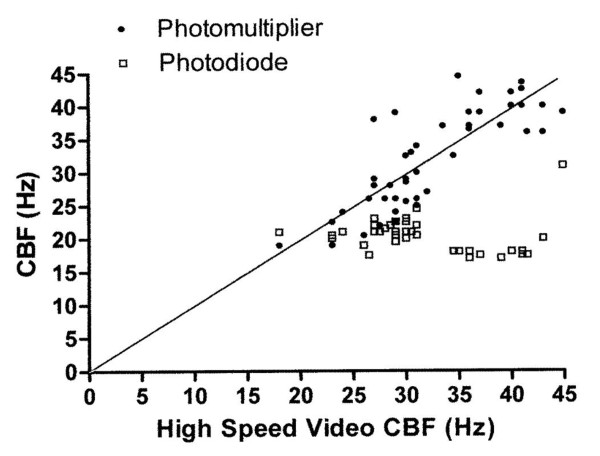
**Ependymal ciliary beat frequency measurements using three different methods**. Readings taken using the photomultiplier and photodiode (y-axis) are plotted against readings from the high speed video (x-axis).

The mean (95% confidence intervals) beat frequencies determined by the high speed video, photomultiplier and photodiode at 30°C were 27.7 (26.6 to 28.8), 25.5(24.4 to 26.6) and 20.8 (20.4 to 21.3) Hz, respectively. At 37°C the differences observed in ciliary beat frequency measurement between all three methods was the greatest. The mean (95% confidence intervals) beat frequencies determined by the high speed video, photomultiplier and photodiode were 36.4(34 to 39.5), 38.4(36.8 to 39.9) and 18.8(16.9 to 20.5) Hz, respectively (Table 1). The individual results are shown in Figure 2 where results from the high speed video are compared with those from the photodiode and photomultiplier.

**Table 1:** Mean (95% confidence intervals) readings of ependymal ciliary beat frequency using three different methods of measurement at two different temperatures

	Frequency (Hz) (95% confidence levels)		
Temperature	HSV	Photomultiplier	Photodiode
30°C	27.7 (26.6 to 28.8)	25.5 (24.4 to 26.6)	20.8 (20.4 to 21.3)
37°C	36.4 (34 to 39.5)	38.4 (36.8 to 39.9)	18.8 (16.9 to 20.5)

HSV and Photomultiplier n = 28 at 30°C and 37°C; photodiode n = 16 at 30°C and n = 23 at 37°C. HSV, high speed video.

Analysis of variance found a highly significant effect of temperature (*P *< 0.001) and method (*P *< 0.001) on the measurement of ciliary beat frequency. A highly significant interaction between method and temperature was observed (*P *< 0.001). The difference in ciliary beat frequency between methods varied at the two temperatures evaluated. The analysis of variance was repeated after omitting the photodiode method. There was still a significant interaction between temperature and method (*P *= 0.003).

As the difference between the methods varies with temperature, the limits of agreement for each temperature and each method were calculated separately. These are shown in Table 2.

**Table 2:** Limits of agreement for comparison to high speed video method

Temperature °C	Method	Difference		Limits of agreement
		Mean	SD	
30	Photomultiplier	2.17	2.60	-3.03 to 7.37
37		-2.00	4.15	-10.30 to 6.29
30	Photodiode	6.58	2.96	0.65 to 12.51
37		18.34	4.94	8.46 to 28.21

The photodiode method underestimates ciliary beat frequency greatly compared with the high speed video. The limits of agreement were narrowest for the photomultiplier method at a temperature of 30°C.

The variance components were remarkably similar even though the number of full cycles measured increased from 5 to 15. This implies that there is little advantage to be gained from increasing the number of cycles measured. The component of variance for intra subject variability was only 1% of the total variation.

Analysis of variance was performed considering the two observers to have fixed effects. This showed significant differences between the means of the two observers (*P *= 0.02). Although there is a significant difference between the two observers, the between observer component of variance is only 3.8% of the total variation.

To evaluate the ciliary beat pattern a total of 106 individual cilia were observed. The results for the angles measured are shown in Table 3. Ependymal cilia were found to beat with a power and recovery stroke within the same plane. Minimal deviation of the cilium from the midline during the power and recovery stroke was observed (Mean angle R = 12.1°, L = 12.6°). No significant difference was found between angle L and R (*P *= 1.0). However, a significant difference was observed between outer left (OL) and outer right (OR) angles (*P *< 0.001).

**Table 3:** Angles measured from slow motion recordings of ependymal cilia (refer to Figure 1).

	N	Mean Angle (°)	SD	SEM	+/_95% Confidence Intervals
L	106	12.1	4.5	0.4	0.9
R	106	12.6	5.1	0.5	1.0
Outer Left	106	85.6	13.4	1.3	2.6
Outer Right	106	69.8	11.8	1.15	2.3

OL: Outer Left: maximum movement of the cilium to the left with reference to the horizontal. OR: Outer Right: maximum movement of the cilium to the right with reference to the horizontal. L: maximum deviation of cilium left of midline. R: maximum deviation of cilium right of midline. N, number; SEM, standard error of the mean.

## Discussion

This study directly compares the most commonly used methods of estimating ciliary beat frequency. The digital high speed video technique is held as the gold standard for the measurement of ciliary beat frequency. A major advantage of the high speed video system is the ability to obtain images at a rate of 400 per second and to make a permanent recording of the ciliary movement at a reduced frame rate. This allows frequency, amplitude and synchrony of ciliary beat to be determined at a later time. The integrity of a ciliated strip can also be assessed during an experiment and the movement of cilia evaluated.

Our results show that the photodiode method is unsuitable for the measurement of cilia beating at high frequencies. There is a broad agreement between the ciliary beat frequency measured by the photomultiplier technique and by high speed video. The limits of agreement are narrowest at a temperature of 30°C, though still large. In addition, the mean measurements made by the photomultiplier at a temperature of 30°C are lower than those made by the high speed video whereas the reverse is true at a temperature of 37°C.

The photodiode technique [14,15] involves the relay of images from a standard video camera to a monitor at a frame rate of 25/second. This explains the very poor agreement between ciliary beat frequency measured by the photodiode technique and the high speed video camera. If cilia are beating faster than 25 frames per second not all of the cycles are detected by the photodiode. The problems encountered with the photodiode may be shared by the analogue contrast enhancement technique which also relies on manipulations of standard video recordings. These techniques may still be suitable for recording the frequency of respiratory cilia which beat at less than 20 Hz. However, pharmacological stimulation which results in an increase of ciliary frequency may not be detected. Although the limits of agreement between photomultiplier readings and high speed video recordings are in a similar range, the readings are different and the techniques cannot be used interchangeably.

The photodiode and photomultiplier techniques are subject to vibration artefact. Readings made directly from the trace of an oscilloscope may include artefacts which may be impossible to differentiate from the true signal. The use of power spectrum analysis gives a more objective measurement of frequency with this method. A spectrum of frequency is obtained which helps to separate out artefactual results. However, it is not always clear which spectrum relates to the ciliary beat cycle [34]. Two closely associated peaks of similar height are sometimes encountered making it difficult to know which to choose.

The photomultiplier technique, initially described by Dalhamn [16], has been widely used to measure ciliary beat frequency [17,18]. The precise method by which the photomultiplier is used varies between authors, particularly regarding the size of the aperture used. While Dalhamn [16] suggests a small aperture of 2.5 microns^2 ^other authors have used large apertures. It is likely that larger apertures may encompass areas where cilia are beating at different frequencies. As previously mentioned one of the major drawbacks of the photomultiplier method is that it does not allow analysis of single cilia or the precise beat pattern to be determined.

The intra subject variation in measurement of ciliary beat frequency from slow motion video tapes was very low. The inter subject variation only accounted for 3.8% of the variability of the system. These results suggest that recordings made may be studied at a later date by a trained observer to facilitate ongoing studies and to check previous results.

Lechtreck *et al*. [29] observed that ependymal cilia showed a relatively planar back and forth motion but did not quantify the movement. The same group have also detailed the methods they used to evaluate ciliary beat frequency, beat pattern and coordination of ependymal cilia from brain slices [35].

The experimental system we utilized allowed the ciliary movement to be observed beating towards the observer. From this we were able to calculate the angle of deviation of the cilium during the power and recovery strokes. We have shown that ependymal cilia not only beat in a planar motion but deviate slightly towards the right during the power stroke and to the left during the recovery stroke. This differs from our results with respiratory cilia that beat forward and backwards with a deviation of only 5° from the perpendicular during their recovery stroke [28]. The reasons for this difference are unclear but ependymal cilia are significantly longer than respiratory cilia and are designed to propel fluid as opposed to mucus. It is also of interest that the ependymal cilia from the wild type mice in Lechtreck's study beat at a lower frequency (10.7 Hz) than tracheal cilia (15.7 Hz) [29]. Although their measurements were made at ambient temperature as opposed to our readings that were made at 37°C we would have predicted that ependymal cilia would beat at higher frequencies and at a much higher frequency than respiratory cilia [13].

## Conclusion

Slow motion replay of high speed video footage of brain ependymal cilia allows both ciliary beat frequency and beat pattern to be assessed. The photodiode technique cannot measure ependymal ciliary beat frequency. It follows that experiments using techniques involving standard video cameras cannot be relied upon to detect either slowing or stimulation of ependymal ciliary beat frequency. The photomultiplier may be used to detect differences in beat frequency at higher frequencies but results still vary considerably from those obtained using a high speed video system. Results from the two systems cannot be directly compared. As opposed to respiratory cilia that beat forwards and backwards with minimal sideways movement, ependymal cilia deviated slightly to the right during their power stroke and to the left during their recovery stroke.

## List of abbreviations

CBF: ciliary beat frequency; CSF: cerebrospinal fluid.

## Competing interests

The authors declare that they have no competing interests.

## Authors' contributions

CO'C conceived the study. MC performed analysis of beat pattern. KS and COC performed beat frequency measurements. CO'C and MC analyzed and interpreted the results and wrote the paper. All authors have read and approved the manuscript.
